# Resilience and psychological factors among dentistry students who received face-to-face lectures during the COVID-19 pandemic

**DOI:** 10.1186/s12909-024-05445-8

**Published:** 2024-04-24

**Authors:** Jesús Rodríguez-Molinero, Inmaculada Corral-Liria, Raquel Jiménez-Fernández, Rosario Ramírez-Puerta, Sara González-Martín, Esther Delgado-Somolinos, Marta Elena Losa-Iglesias, Antonio Francisco López-Sánchez

**Affiliations:** 1 Department of Nursing and Stomatology, Faculty of Health Sciences, Rey Juan Carlos University, 28922 Alcorcón, Madrid, Spain; 2 IDIBO Research Group, Rey Juan Carlos University, Alcorcón, Madrid Spain; 3 IDRENF Research Group, Rey Juan Carlos University, Alcorcón, Madrid Spain

**Keywords:** Anxiety, Depression, Resilience, Dentistry, Students, Learning

## Abstract

**Background:**

This research evaluated whether the relationships between factors of resilience, self-esteem, depression, and anxiety in dental students with changes in teaching and learning methods. We also studied the psychological impact of face-to-face lectures during the COVID-19 pandemic.

**Methods:**

This cross-sectional descriptive study used Google Forms to collect data with the Rosenberg Self-Esteem Scale (RSE), Connor-Davidson Risk Resilience Scale (CD-RISC), Beck Anxiety Inventory (BAI), and Beck Depression Inventory (BDI and BDI-II). An open-ended question was also asked about important learning difficulties.

**Results:**

The analysis revealed very high levels of resilience (30.23 ± 5.84), self-esteem in the normal range (29.08 ± 4.03), minimal depression levels (12.32 ± 8.05), and low anxiety levels (17.20 ± 12.41). There were no significant differences between sociodemographic variables ranges in regard to all psychological questionnaires. No high levels of depression and anxiety were found.

**Conclusions:**

The levels were low compared to other studies in which online teaching was used, which is explained by the fact that the students retained adequate resilience and self-esteem thanks to being able to contact teachers and, above all, their own peers.

## Introduction

At the end of 2019 and beginning of 2020, a new upper-respiratory infection appeared in Wuhan, China. The pathogen was a new type of highly contagious coronavirus, SARS-CoV-2 (severe acute respiratory syndrome coronavirus 2). The disease it caused is called COVID-19 (coronavirus disease 2019) [1]. The rapid spread of the virus forced the World Health Organization (WHO) to declare a pandemic status in early March 2020 [2].

In response, several countries, including Spain, adopted restrictive measures aimed at reducing close contact between people and stopping the spread of the virus through quarantine and isolation strategies [3]. These measures also affected academic institutions and especially dental schools due to the high risk of students in this health profession for exposure to contaminated material and transmission through aerosols [4]. During the time in which in-person teaching was interrupted, online teaching strategies were implemented and posed a challenge for both students and teachers [5]. The initial teaching models in many universities were online, in which students were isolated and classes could not be taught face-to-face. As the health situation improved, hybrid teaching models were implemented, in which half of the students could attend in person, while others did so online [6].

However, the specific characteristics of the dental profession require dental students to develop skills in a clinical environment and in person, so not being able to attend classes and practice normally could be a factor in restlessness and stress related to graduating and subsequent unemployment [7]. Although the overload of stress and difficulties of dental students have been the subject of study [8], the appearance of COVID-19 and its implications in the interruption of studies, the adaptation to new teaching models, and the reduction of clinical practice in the last years of training have generated new destabilizing factors that are of interest for analysis [9].

In many universities and university programs, the number of students was too high to maintain sanitary measures that guaranteed the safety of the students [10]. Despite this, in some centres with a very small number of dental students, face-to-face teaching could be maintained with appropriate health measures. This fact is of special interest for analysing the extent to which in-person teaching has affected the students during the pandemic period, in which the majority of healthcare students in other degrees had online or hybrid classes. For these reasons, the main objective of this research was to evaluate whether there is a relationship between factors such as resilience, self-esteem, depression, and anxiety in dental students due to changes in teaching and learning methods. We also studied the possible psychological impact on these students of face-to-face lectures during the COVID-19 pandemic.

## Methods

### Design and setting

A cross-sectional descriptive study was done with non-random sampling of dentistry students who had face-to-face learning during the COVID-19 pandemic. The data were collected using Google Forms which included consent, demographic variables (age, gender, marital status, weight, height, occupation, children, dependents, and whether they had COVID-19 at the time of the study), and selected questionnaires: (1) the Rosenberg Self-Esteem Scale (RSE) [11, 12], (2) the Connor-Davidson Risk Resilience Scale (CD-RISC) [13, 14], (3) the Beck Anxiety Inventory (BAI) [15], and (4) the Beck Depression Inventory (BDI and BDI-II) [16]. Finally, an open-ended question was asked about learning difficulties that were important to the respondent.

### Sample

Participants were dental students at the University of Rey Juan Carlos in Spain. The study was conducted from October 1, 2021, to November 29, 2021. The sample size was calculated with software from *Unidad de Epidemiología Clínica y Bioestadística, Complexo Hospitalario Universitario de A Coruña, Universidade A Coruña* (www.fisterra.com). The final number of participants needed was calculated as 92 from a sample of 120 people with an alpha error of 0.05, a confidence interval (CI) of 95%, and 50% variance. The final sample consisted of 92 dental students. The inclusion criteria were (1) dental students from University of Rey Juan Carlos and (2) adequate knowledge of the Spanish language in oral and written form. The exclusion criterion was not full filling out the survey.

### Assessment scales

#### Rosenberg Self-Esteem Scale (RSE)

The RSE questionnaire consists of 10 questions scored from 1 to 4 (4 = strongly agree, 3 = agree, 2 = disagree, 1 = strongly disagree). Five statements are positive, and five statements are negative. The survey authors set limits for this scale, and a score of 20 to 30 is generally considered normal. A score above the normal range indicates high self-esteem, while a result below the normal range indicates low self-esteem. The RSE has been shown to be positively correlated for both men and women across ethnic groups [17]. The scale is reliable with test–retest correlations in the range of 0.82 to 0.88 [11] and 0.87 for the Hispanic population [12].

### Connor-Davidson Risk Resilience Scale (CD-RISC)

Resilience was assessed using the abbreviated version of the CD-RISC, which was validated in Spanish by Notario-Pacheco et al*.* [14]. The scale consists of 10 items (items 1, 4, 6, 7, 8, 11, 14, 16, 17, and 19 from the original scale developed by Connor et al*.* [13]). Using this measure, participants were asked to respond about the extent to which they agree with each statement presented to them (for example, Item 1: "I can change it"). The response is a five-point Likert scale ranging from 0 (strongly disagree) to 4 (strongly agree). Resilience was defined with a weighted scale of 0.48 to 0.76 and a Cronbach's alpha of 0.85 [14].

### Beck Anxiety Inventory (BAI)

The BAI uses a list of 21 symptoms of anxiety rated on a 4-point Likert scale with results ranging from not being anxious to being severely anxious. The inventory shows how much each symptom has affected the respondent in the past week. The values ​​of each score are summed up, and a total score between 0 and 63 points is obtained. A total score of 0–7 is defined as the lowest anxiety level, 8–15 is mild, 16–25 is moderate, and 26–63 indicates severe anxiety [18].

In a Mexican population, the scale had good correlation with a Cronbach's alpha coefficient of 0.92 and test–retest reliability of 0.75. The internal consistency of the BAI is high (Cronbach's alpha is 0.90 to 0.94). The correlation between each item and the total score is between 0.30 and 0.71. The test–retest reliability ranged from 0.67 to 0.93 after one week and was 0.62 after 7 weeks [19].

### Beck Depression Inventory (BDI and BDI-II)

The BDI is a 21-item scale with all questions answered on a Likert scale. The internal consistency measure alpha is 0.78. Examples of items for topics such as sadness include "I'm often sorry" or "I'm not sad". The original BDI-II [16] proposed the following cut-off values and corresponding depression scales: 0–13 for minimal depression, 14–19 for mild depression, 20–28 for moderate depression, and 29–63 for severe depression. The Spanish version by Sanz et al*.* [20] used the cut-off scores of Beck et al*.* [16], and they concluded that the reliability of the instrument was high in terms of consistency (Cronbach's alpha coefficient = 0.83) and reliability (test–retest correlations of 0.60 and 0.72 for three different groups from all samples).

### Ethical considerations

All participants confirmed their eligibility before filling out the questionnaire. This study was approved by the Rey Juan Carlos University Ethics Committee (number: 2910202121221).

### Data analysis

All variables were tested for normal distribution using the Kolmogorov–Smirnov test, and data were considered normally distributed at p > 0.05. Analysis was performed using the mean and variance for multiple variables and using counts and percentages for differences. Spearman correlation was used to evaluate the strength of the relationship between variables, and Pearson correlation and the Mann–Whitney U test were used to analyse the relationship between variables. All statistics were considered significant at *p* < 0.05 (SPSS for Windows, version 20.0; SPSS Inc., Chicago, IL).

We reviewed the open-ended question using the program Nvivo™ 8 to explain the answers, findings, and interpretation of the digital pressure graph. Answers were analysed in several stages, including selecting terms, grouping them according to lexical criteria, creating groups and categories, and establishing word clouds. The clouds formed a spiral layer, and most of the answers appeared in capital letters and in the middle of the cloud.

## Results

Different variables showed non-normal and normal distributions, as shown in Table 1. The analysis of the descriptive data of each scale revealed very high levels of resilience (30.23 ± 5.84), self-esteem in the normal range (29.08 ± 4.03), minimal depression levels (12.32 ± 8.05), and low anxiety levels (17.20 ± 12.41). As shown Tables 2, 3, 4 and 5, we did not find significant differences between sexes, socioeconomic statuses, way of living (alone, with family, etc.), and age ranges regarding all psychological questionnaires.

**Table 1:** Descriptive data of the participants

**Descriptive Data**	**Total Group** **Mean ± SD** **(95%CI)** ***N* = 92**	**Male** **Mean ± SD** **(95%CI)** ***n* = 19**	**Female** **Mean ± SD** **(95%CI)** ***n* = 73**	*p*-value*
Age (years)	22.65 ± 4.85 (21.66–23.64)	23.11 ± 7.50 (19.73–25.48)	22.53 ± 3.95 (21.63–23.44)	0.650
Weight (kg)	57.59 ± 10.18 (55.51–59.67)	68.05 ± 10.98 (63.11–72.99)	54.86 ± 8.03 (53.02–56.71)	< 0.001
Height (cm)	164.90 ± 8.15 (163.24–166.57)	173.95 ± 6.96 (170.82–177.08)	162.55 ± 6.68 (161.02–164.08)	< 0.001
BMI (kg/m^2^)	21.06 ± 3.14 (20.42–21.70)	22.55 ± 3.83 (20.83–24.27)	20.67 ± 2.83 (20.02–21.32)	0.520

*Abbreviations*: *BMI* Body mass index, *kg* kilograms, *m* meters, *SD* Standard deviation, *CI* confidence interval. * Independent t student were applied. In all analyses, p < 0.05 (with 95% CI) was considered statistically significant

**Table 2:** Descriptive data based on different assessment scales between sexes

**Scale**	**MALE** **Mean (SD)** **(CI 95%)** ***n* = 19**	**FEMALE** **Mean (SD)** **(CI 95%)** ***n* = 73**	***p*-value**
CD-RISC	31.10 ± 5.85 (28.47–33.73)	30.01 ± 5.85 (28.67–31.35)	0.471*
BECK (BDI-II)	11.52 ± 8.03 (7.91–15.14)	12.53 ± 8.10 (10.67–14.39)	0.629*
BECK (BAI)	13.89 ± 9.84 (9.46–18.32)	18.46 ± 12.89 (15.50–21.42)	0.154**
ROSEMBERG SELF-STEEMRSE	28.94 ± 4.07 (29.11–30.77)	29.12 ± 4.05 (28.19–30.05)	0.866**

*Abbreviations*: *SD* Standard deviation, *CI* Confidence interval. * Independent Student t-test. ** U Mann Whitney. In all analyses, *p* < 0.05 (with 95% CI) was considered statistically significant

**Table 3:** Descriptive data based on different assessment scales between socioeconomic statuses

**Scale**	**MEDIUM–HIGH LEVEL** **Mean (SD)** **(CI 95%)** ***n* = 87**	**LOW LEVEL** **Mean (SD)** **(CI 95%)** ***n* = 5**	***p*-value**
CD-RISC	30.45 ± 5.38 (29.32–31.59)	26.40 ± 11.50 (16.31–36.48)	0.131*
BECK (BDI-II)	12.25 ± 8.19 (10.53–13.97)	13.60 ± 5.59 (8.69–18.50)	0.718*
BECK (BAI)	17.64 ± 12.58 (14.99–20.28)	15.40 ± 9.98 (6.64–24.15)	0.696**
ROSEMBERG SELF-STEEMRSE	29.01 ± 4.08 (28.15–29.86)	30.40 ± 3.20 (27.58–33.21)	0.457**

*Abbreviations*: *SD* Standard deviation, *CI* Confidence interval. * Independent Student t test. ** U Mann Whitney test. In all analyses, *p* < 0.05 (with 95% CI) was considered statistically significant

**Table 4:** Descriptive data based on different assessment scales for way of living (alone, with family, etc.)

**Scale**	**ALONE** **Mean (SD)** **(CI 95%)** ***n* = 7**	**ACCOMPANIED** **Mean (SD)** **(CI 95%)** ***n* = 85**	***p*-value**
CD-RISC	27.57 ± 10.35 (19.89–35.24)	30.45 ± 5.35 (29.32–31.59)	0.210*
BECK (BDI-II)	15.00 ± 7.87 (9.16–20.83)	12.10 ± 8.07 (10.38–13.82)	0.363*
BECK (BAI)	19.14 ± 10.35 (11.47–26.81)	17.38 ± 12.61 (14.70–20.07)	0.721**
ROSEMBERG SELF-STEEMRSE	29.01 ± 4.08 (28.15–29.86)	30.40 ± 3.20 (27.58–33.21)	0.457**

*Abbreviations*: *SD* Standard deviation, *CI* confidence interval. * Independent Student t test. ** U Mann Whitney test. In all analyses, *p* < 0.05 (with 95% CI) was considered statistically significant

**Table 5:** Descriptive data based on different assessment scales between age ranges (18 to 21 years versus 22 to 52 years)

**Scale**	**18 to 21 years old** **Mean (SD)** **(CI 95%)** ***n* = 47**	**22 to 52 years old** **Mean (SD)** **(CI 95%)** ***N* = 45**	***p*-value**
CD-RISC	29.42 ± 4.47 (28.14–30.70)	31.08 ± 6.94 (29.06–31.45)	0.173*
BECK (BDI-II)	15.00 ± 7.87 (9.16–20.83)	12.10 ± 8.07 (10.38–13.82)	0.075*
BECK (BAI)	18.87 ± 12.38 (15.33–22.41)	16.11 ± 12.43 (12.47–22.50)	0.289**
ROSEMBERG SELF-STEEM	13.78 ± 7.70 (11.58–15.99)	10.80 ± 8.21 (8.39–16.18)	0.109**

*Abbreviations*: *SD* Standard deviation, *CI* Confidence interval. * Independent Student t test. ** U Mann Whitney test. In all analyses, p < 0.05 (with 95% CI) was considered statistically significant

The analysis showed significant negative correlations between CD-RISC, BDI-II, and BAI (*p* < 0.001). In addition, the RSE showed significant negative correlations with the BDI-II and BAI (*p* < 0.001). Finally, there were significant positive correlations between the BDI-II and BAI and between the RSE and CD-RISC (*p* < 0.001), as shown in Table 6.

**Table 6:** Correlation and differences between scales (Spearman’s correlation coefficient (p-value))

Scale	CD-RISC	BECK (BDI- II)	BECK (BAI)	ROSEMBERG SELF-STEEM
CD-RISC	1			
BECK (BDI- II)	− 0.372 (< 0.001)	1		
BECK (BAI)	− 0.441 (< 0.001)	0.694 (< 0.001)	1	
ROSEMBERG SELF-STEEM	0.557 (< 0.001)	− 0.600 (< 0.001)	− 0.473 (< 0.001)	1

Statistical significance for *p* < 0.05 with 95% CI

### Open question

**Fig. 1:**
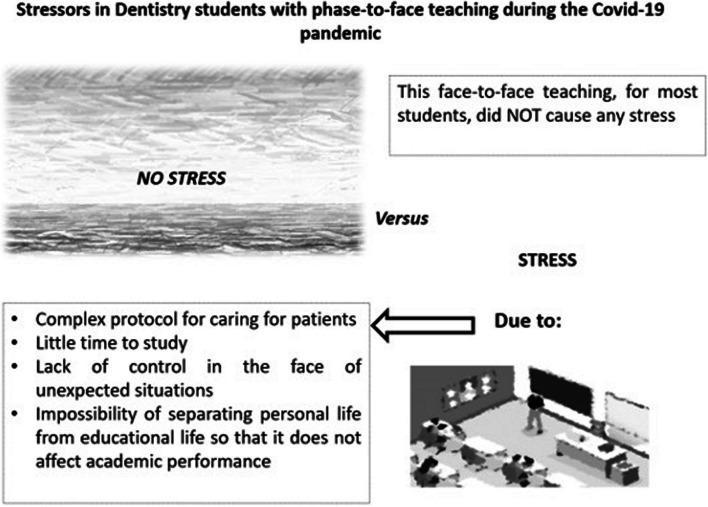
Stressors in dentistry students with face-to-face teaching during the COVID-19 pandemic

The open-question responses shared emotions and experiences. The main topic was that the students did not have stress or academic stress at all. The few students that had written feelings wrote about the complex protocol of caring for patients, little time to study, lack of control in the face of unexpected situations, and the impossibility of separating personal life from educational life so that it does not affect academic performance (Fig. 1).

## Discussion

This study examined how modifications in teaching methods could have affected the levels of stress, anxiety, depression, and resilience of dental students and whether there was any psychosocial impact on them during full face-to-face teaching during the COVID-19 pandemic. The results showed that the dental students had high levels of resilience along with low levels of anxiety in normal ranges. The results are similar to those of German and Saudi studies, in which levels of anxiety, stress, and depression were normal or mild [21, 22]. However, most studies show high levels that even reach depression values at rates of 60.7% and 75.3% [23, 24].

Başağaoğlu et al. compared stress and anxiety levels in dental students who received online instruction versus those who did face-to-face classes [25]. High levels of both stress and anxiety were found in the first group, but no statistically significant differences were found with the group that received face-to-face instruction. The disparity in results between studies may be due to cultural differences [26] or differences in teaching models that existed before the COVID-19 pandemic [27].

Regarding self-esteem, in a study carried out in the USA, a direct relationship with stress was found in dental students. High levels of stress were associated with significant self-esteem problems, especially in students between the ages of 25 and 34 and those who were further away from finishing their education [28]. In our study, resilience and empathy were protective factors against anxiety and depression, regardless of gender or academic year. Regarding sociodemographic variables, a greater association between higher levels of depression and female sex has been observed [27]. However, we did not find data in our results that indicate a higher prevalence in terms of sex.

Age was also not indicative of higher levels of depression or anxiety. Only one study compared the levels between students and dentists who have graduated. Higher levels of depression were found in the student group, but there were no statistically significant differences between the two groups [29].

One of the limitations of this study is that there was a small sample of dentistry students due to the small number of dental students who enter the university each year. It would be interesting to compare similar studies in other universities where dentistry is studied. However, the teaching models are not the same, and it is impossible to obtain comparable samples.

## Conclusions

In the present study, high levels of depression and anxiety were not found in dental students who had face-to-face teaching during the COVID-19 pandemic. The level of depression and anxiety was low compared to other studies in which online teaching was used. This is explained by the fact that students retain adequate resilience and self-esteem thanks to being able to have contact with teachers and especially with their own classmates. Due to these results, we consider that if we had to return to a situation similar to the COVID-19 pandemic, we should maintain face-to-face teaching with small groups as long as the health situation allows it in order to reduce the levels of depression and anxiety of students.

## Data Availability

All data generated or analysed during this study are included in this published article [and its supplementary information files].
